# Establishment and application of a real-time loop-mediated isothermal amplification system for the detection of *CYP2C19* polymorphisms

**DOI:** 10.1038/srep26533

**Published:** 2016-06-01

**Authors:** Chao Zhang, Yao Yao, Juan-Li Zhu, Si-Nong Zhang, Shan-Shan Zhang, Hua Wei, Wen-Li Hui, Ya-Li Cui

**Affiliations:** 1 College of Life Sciences, Northwest University, Xi’an, 710069, China; 2 National Engineering Research Center for Miniaturized Detection Systems, Xi’an, 710069, China

## Abstract

Single-nucleotide polymorphisms (SNPs) represent the most widespread type of genetic variation (approximately 90%) in the human genome, and the demand to overcome such variation has received more attention now than ever before. The capacity to rapidly assess SNPs that correlate with disease predisposition, drug efficacy and drug toxicity is a key step for the development of personalized medicine. In this work, a rapid one-step SNP detection method, real-time loop-mediated isothermal amplification (RT-LAMP), was first applied for *CYP2C19* polymorphisms testing. The optimized method was established with specifically designed primers for target amplification by real-time detection in approximately 30 min under isothermal conditions. RT-LAMP amplified few copies of template to produce significant amounts of product and quantitatively detected human DNA with compatible specificity and sensitivity. The success in the establishment of this RT-LAMP protocol for *CYP2C19* polymorphism testing is significant for the extension of this technique for the detection of other SNPs, which will further facilitate the development of personalized medicine.

Clinical observations beginning in the 1950s have suggested that individuals exhibit differences in their responses to drugs and that these variations could be inherited^1^. The detection of DNA sequence variations provides valuable insight into the diagnosis of genetic-related diseases and conditions, especially for early-stage treatment and response monitoring^2^. Thus, it is critically important to select a method with high sensitivity and specificity to detect single or small numbers of nucleotide polymorphisms^3^,^4^.

Current detection methods rely on sample amplification combined with meticulous control, including polymerase chain reaction (PCR), nucleic acid sequence-based amplification (NASBA)^5^, self-sustained sequence replication (3SR)^6^, strand displacement amplification (SDA)^7^ and direct sequencing. Although these technologies are currently considered the gold standard for laboratory-based DNA detection and diagnostics, these methods cannot meet the requirements of point-of-care testing (POCT) strategies^8^ due to the high set up and operating expenses and the requirements for high-precision equipment. Loop-mediated isothermal amplification (LAMP) is an outstanding gene amplification procedure with high specificity, sensitivity and rapidity that was established by Notomi *et al*.^9^. The process amplifies nucleic acids under isothermal conditions and employs self-recurring strand-displacement synthesis primed by a specially designed set of target-specific primers^10^, thus clearly distinguishing this technique from existing genetic tests^11^.

LAMP is characterized by the use of six specifically designed primer regions to recognize eight regions on the target DNA; thus, the specificity is extremely high^11^. Amplification and detection of a gene can be completed in a one step by incubating the mixture of sample, primers, DNA polymerase with strand displacement activity and substrates under isothermal conditions between 60 and 65 °C. LAMP has been used for the diagnosis of pathogens *via* the detection of gene segments, e.g., the diagnosis of infectious diseases, such as Japanese encephalitis virus infection^12^, rapid genotyping of carcinogenic human papillomavirus and herpesvirus^12^, and the detection of Middle East Respiratory Syndrome Coronavirus^12^,^13^,^14^. Numerous investigations have demonstrated that this special identification system is more accurate than PCR-based methods, which use only two primers to recognize two regions.

Although detection of human DNA polymorphisms using LAMP is challenging, especially for single-nucleotide polymorphisms (SNPs) due to the complex nature of DNA compared with microbes and viruses^2^, SNPs represent the most widespread type of genetic variation (approximately 90%) in the human genome^15^, and the capacity to rapidly test patients for SNPs that are correlated with disease predisposition, drug metabolism and disease development is a key step for the development of personalized medicine^16^. Thus, the wide application of this simple, rapid and low-cost genotyping LAMP method in SNP detection is imperative.

Numerous lines of evidence have strongly suggested that genetic polymorphisms in drug-metabolizing enzymes, transporters, receptors and other drug targets are associated with inter-individual differences in drug treatment response^17^. Sequence variations in drug target proteins, drug-metabolizing enzymes, and drug transporters can alter drug efficacy, drug side effects, or both to cause variable drug responses in individual patients^18^. For example, on March 12, 2010, the US Food and Drug Administration approved a black box warning regarding the diminished effectiveness of clopidogrel in patients who carry two loss-of-function alleles (poor metabolizers)^19^, i.e., CYP2C19*2 (G681A) and CYP2C19*3 (G636A) alleles, which account for 85% and 99% of the nonfunctional alleles in Whites and Asians, respectively^20^,^21^. The warning addressed the need for polymorphism genotyping to identify altered clopidogrel metabolism in patients^22^.

We developed a rapid, one-step SNP detection method (RT-LAMP) that enables the detection of the *CYP2C19* allele in approximately 30 min under isothermal conditions. The optimized RT-LAMP technique is more suitable for point-of-care testing and will further facilitate on-site screening. The successful establishment of an inexpensive, rapid and real-time LAMP protocol for CYP2C19*2 and CYP2C19*3 detection is significant for the extension of this technique for genotyping other SNPs. Our results suggest applications for this RT-LAMP assay system for both basic research and clinical diagnosis in pharmacogenomics.

## Results

### Plasmid construction and identification

In this study, four plasmids were constructed by recombining the specific sequences of CYP2C19*2 G681G, CYP2C19*2 A681A, CYP2C19*3 G636G and CYP2C19*3 A636A. Using the primer pairs *2-seq-F/*2-seq-R and *3-seq-F/*3-seq-R, listed in Table 1, 226-bp CYP2C19*2 and 450-bp CYP2C19*3 fragments were amplified and sequenced by Beijing Genomics Institute (BGI; Beijing, China), indicating the successful incorporation of the four plasmids (data not shown).

### Optimization of the RT-LAMP primers

**Figure 1:**
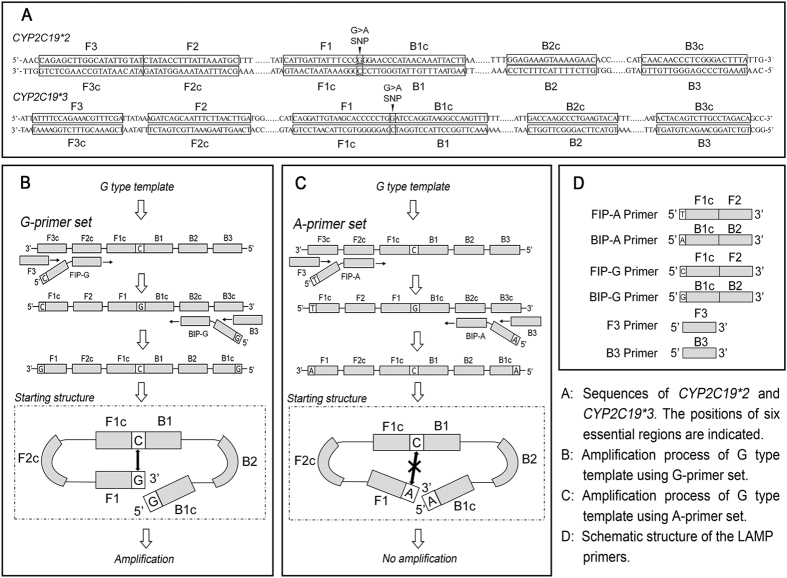
Schematic of primers and the RT-LAMP-based SNP detection process.

**Figure 2:**
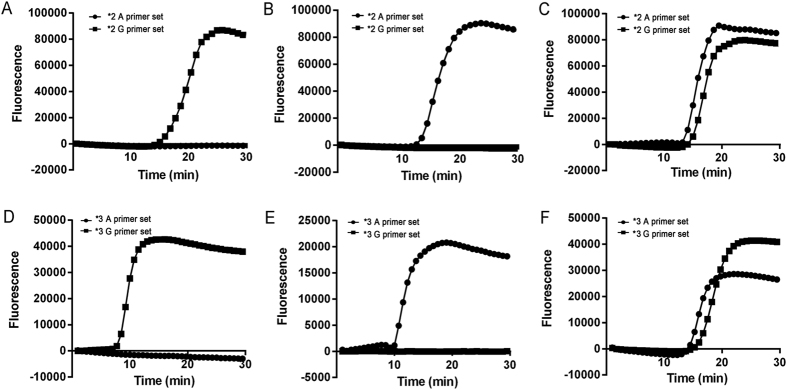
RT-LAM-based detection of CYP2C19*2 and CYP2C19*3. (**A–C**): using CYP2C19*2 G681G, CYP2C19*2 A681A and mixed plasmids as templates, respectively; (**D–F**): using CYP2C1*3 G636G, CYP2C19*3 A636A and mixed plasmids as templates, respectively. The following genotypes were identified: CYP2C19*2 G681G, CYP2C19*2 A681A, CYP2C19*2 G681A, CYP2C19*3 G636G, CYP2C19*3 A636A, and CYP2C19*3 G636A.

Based on the point mutations of the CYP2C19*2 (G681A) and CYP2C19*3 (G636A) genes, two sets of RT-LAMP primers were designed to discriminate the SNPs. As shown in Fig. 1, the basic principle of RT-LAMP involves the use of specific primers, with a forward inner primer (FIP) and backward inner primer (BIP) that are designed to contain a SNP nucleotide at the 5′ terminus, each reaction including two common primers (F3 and B3) and two specific primers (FIP-G and BIP-G for G allele and FIP-A and BIP-A for A allele). The structures of the LAMP primers and products based on this study are presented in Fig. 1, and the information regarding the primer names and sequences are provided in Table 1. The target SNP was characterized using six different regions (F1/F1c-F3/F3c and B1/B1c-B3/B3c) specifically designed to recognize distinct regions on the target gene, which were designed to ensure that the primers would specifically amplify the G681A and G636A substitutions. The results depicted in Fig. 2 illustrate that the RT-LAMP method could accurately detect and discriminate all possible homozygotes and heterozygotes of CYP2C19*2 (G681A) and CYP2C19*3 (G636A) SNPs.

### Optimization of the RT-LAMP reaction

When RT-LAMP was performed using the four plasmids as the templates, the best results were obtained in a final reaction volume of 25 μL. The reaction mixture contained 15 μL of Isothermal Master Mix (OptiGene Ltd., UK) containing *Geobacillus* DNA polymerase, thermostable inorganic pyrophosphatase, optimized buffer including MgCl_2_, dNTPs and ds-DNA dye, 5 μL of primer mix consisting of four primers each for F3 and B3 primers at 0.2 μM, FIP and BIP primers at 0.4 μM, and 5 μL ddH_2_O. The reaction was performed in 8-well 0.2-mL tubes with incubation at 62 °C (decided based on a series temperature gradient test) for 30 min. The fluorescence intensity of the ds-DNA dye was simultaneously monitored in a real-time fluorometer (Genie II from OptiGene Ltd. UK).

### Sensitivity of the RT-LAMP assay

**Figure 3:**
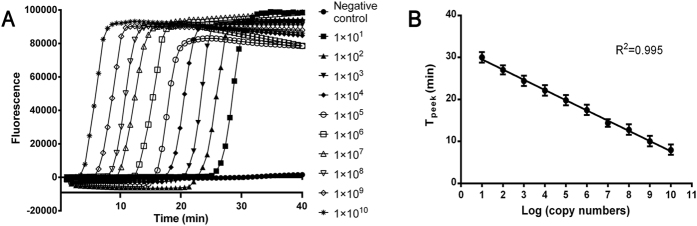
Sensitivity of the RT-LAMP assay using *CYP2C19* G681G plasmid. (**A**) Sensitivity of the RT-LAMP assay as monitored using the Genie II system. (**B**) The standard curve was generated from a dilution series of plasmid by plotting the T_peek_ versus the plasmid copy number.

The sensitivities of the RT-LAMP assay were tested using 10-fold serial dilutions of the four constructed plasmids. The detection limit of the RT-LAMP assay was 1 × 10^1^ copies of plasmid (the result for the *CYP2C19* G681G plasmid is presented in Fig. 3A; the results for the other three plasmids are presented in supplementary Fig. S1), indicating that the RT-LAMP method was efficient and specific in SNP detection under a constant temperature with greater than 10-fold increased sensitivity compared with conventional PCR^23^,^24^. Moreover, as shown in Fig. 3, a standard curve was generated using 10-fold dilutions of plasmids and calculated by regression analysis comparing the T_peek_ with the copy number. The high correlation coefficient (R^2^ = 0.995) indicated that the RT-LAMP assay could be applied in DNA quantification.

### Evaluation of RT-LAMP using clinical samples

To test the reliability of the RT-LAMP system optimized in this study, the accuracy of the RT-LAMP assay was further verified using 100 clinical samples. In addition, all of these samples were also assessed *via* conventional PCR (AS-PCR) and sequenced by BGI. The detected genotypes together with their frequencies are presented in Table 2. The observed allele frequencies were 75.5%, 24.5%, 95% and 5%, for *2G, *2A, *3G and *3A, respectively (calculated from: *2G: F1 + F3 + F4/2 + F5 + F6/2; *2A: F2 + F4/2 + F6/2; *3G: F1 + F2 + F4 + F5/2 + F6/2; *3A: F3 + F5/2 + F6/2). The frequencies of the CYP2C19*2 and CYP2C19*3 alleles were similar to those reported by Chen *et al*.^25^ in a Chinese population. The comparison of RT-LAMP with AS-PCR and direct sequencing revealed no discrepancies.

## Discussion

In recent years, DNA testing technology has been extensively used in the areas of diagnosis and disease detection. The LAMP technique is a unique assay with high efficiency and high accuracy that was developed rapidly. As described in some reports^10^,^11^,^12^,^13^,^14^,^26^,^27^,^28^,^29^, the LAMP assay has been widely used in the detection of pathogenic microorganisms. However, to the best of our knowledge, reports regarding the detection of mutations in human genomic DNA, especially SNPs, using the LAMP method are lacking. In this study, the successful establishment of an inexpensive, rapid and real-time LAMP protocol for *CYP2C19* SNP genotyping expanded the scope of application of this technique to human gene mutation detection.

RT-LAMP is a one-step method wherein the amplification itself is the signal for SNP detection, whereas the difficulty in developing this technology involves the suppression of non-specific amplification^30^. To help overcome this difficulty, the target SNP is characterized using six different primer regions specifically designed to recognize eight distinct regions on the target gene^14^,^30^. In this work, by adding or subtracting a few nucleotides in the primer regions, the T_m_ value and GC content were calculated until the six different primer regions were suitable for the LAMP reaction. As a result, the primer regions were selected as noted in Fig. 1 (F1-F3 and B1-B3). Furthermore, as the arrangement and composition of human genomic DNA is very complex, sequence alignment was necessary to avoid false recognition of the specific site. Thus, Primer-BLAST software (http://www.ncbi.nlm.nih.gov/tools/primer-blast) was used to ensure that the chosen primer regions were specific to the target SNP, which helped avoid mismatches and locate the target SNP as accurately as possible.

The secondary structures of these primers may cause non-specific results in the LAMP reaction given that the selectable sequence area for primer design is limited to less than four hundred nucleotides surrounding the target SNP site^9^. Hence, the inner primers were designed (Fig. 1) to minimize the impact of the secondary structure given that the inner primers are the main component for DNA strand extension. Moreover, according to Tomita *et al*., the formation of a starting structure is the key initiating step of LAMP^11^. Specific nucleotides were added to the 5′ termini (5′-term) of the FIP and BIP primers to establish a complete and effective starting structure, as shown in Fig. 1. Consequently the starting structure would be successfully established only when the 5′-term nucleotide was exactly matched with the target SNP (Fig. 1A). Otherwise, the starting structure was blocked, as shown in Fig. 1C, and the amplification could not proceed. In conclusion, non-specific amplification was effectively suppressed in this work through special primer design and accurate target location. In addition, using real-time fluorescence detection equipment, amplification and detection can be performed in a closed tube, which could reduce the risk of contamination.

Thus, the RT-LAMP method that was developed has an excellent sensitivity for detection of *CYP2C19* polymorphisms (as shown in Fig. 3A). Similar results were observed by Singh *et al*.^23^ and Lee *et al*.^35^ with the same sensitivity of 1 × 10^1^ copies. Moreover, a standard curve with a high correlation coefficient was obtained in this study, as shown in Fig. 3B, indicating that except for microorganism quantification^32^,^33^,^34^, the RT-LAMP assay can also be applied in human DNA quantification. Therefore, the RT-LAMP method can be used for the determination of trace amounts of DNA of interest among copious background DNA, such as specific mutation detection in circulating tumour DNA^24^,^35^, and can be applied for complex gene quantification, which is clinically meaningful^36^,^37^.

In summary, as a rapid, feasible and cost-efficient point-of-care (POC) SNP detection method, we demonstrated that RT-LAMP could quantitatively detect human genomic DNA with high specificity and sensitivity in a single step. Moreover, the LAMP method can amplify few copies of template to significant levels in 30 min^11^ and can be used for both DNA and RNA targets^38^. Thus, this POC detection method should be helpful in basic research in a variety of fields, including medicine, pharmaceuticals, environmental hygiene, food security, and pharmacogenomics testing.

## Methods

### Peripheral blood and genomic DNA extraction

Peripheral blood samples were collected from 100 unrelated Chinese volunteers using EDTA-coated tubes at the Shaanxi Provincial People’s Hospital (Xi’an, China) with informed consent. The study was approved by the ethics committee of the National Engineering Research Center for Miniaturized Detection Systems, Xi’an, China. All methods were performed in accordance with these approved guidelines. The genomic DNA from the volunteer was isolated from 200 μL of blood using a Whole Blood Genomic DNA Isolation Kit (Xi’an GoldMag Nanobiotech Co., Ltd., Xi’an, Shaanxi, China), according to the manufacturer’s instructions. The final DNA quality and concentrations were measured using a NanoDrop 2000c/2000 UV-Vis spectrophotometer (Thermo Fisher Scientific, Wilmington, DE, USA), according to the manufacturer’s instructions.

### Primer design and synthesis

For each SNP, six primers for RT-LAMP were designed, including two outer primers (common primers, F3 and B3) and four inner primers (specific primers, FIP-G, FIP-A, BIP-G and BIP-A) that recognize distinct regions of the CYP2C19*2 and CYP2C19*3 alleles (rs4244285 and rs4986893). Conventional PCR primers were designed based on the principle of AS-PCR using the Primer 5.0 software program (Primer-E Ltd., Plymouth, UK). All oligonucleotide primers were synthesized by Invitrogen Biotechnology Ltd. (Shanghai, China).

### Plasmids for CYP2C19*2 and CYP2C19*3

Plasmids 19-2GG, 19-2AA, 19-3GG and 19-3AA, which contain the CYP2C19*2 G681G, CYP2C19*2 A681A, CYP2C19*3 G636G, and CYP2C19*3 A636A genes, respectively, were constructed using the pMD^TM^19-T Vector Cloning Kit (Takara Bio Inc. Dalian, China) and were extracted from transformed *Escherichia coli* DH5α cells using a TIANprep Mini Plasmid Kit (Tiangen Biotech Co., Ltd. Beijing, China). The plasmids were identified *via* sequencing by Beijing Genomic Institute (BGI, Beijing, China). The concentrations were determined using a NanoDrop 2000c/2000 UV-Vis spectrophotometer (Thermo Fisher Scientific, Wilmington, DE, USA).

### Optimization of RT-LAMP reaction

The initial condition of the RT-LAMP reaction was adopted from Zhang *et al*.^39^. The LAMP reaction mixtures were incubated for 45 min at 60, 61, 62, 63, 64, or 65 °C to determine the optimal reaction temperature. Then, the LAMP reaction was performed at the optimal reaction temperature for 15, 30, 45, 60, and 90 min to determine the optimal reaction time.

### Conventional PCR reaction

For optimized AS-PCR, the reactions were performed using final volumes of 50 μL, including 25 μL 2  ×  Taq MasterMix (containing Taq DNA polymerase, dNTPs, Mg^2+^, and Taq reaction buffer, Tiangen Biotech Co., Ltd. Beijing, China), 0.4 μM each primer, and 1.0 μL template DNA. All of the amplifications were performed using a 2720 Thermal Cycler (Applied Biosystems, Foster City, CA, USA) with the following parameters: one step of 5 min at 95 °C; 32 cycles of 30 s at 95 °C, 30 s at 60 °C, 45 s at 72 °C; and one step of 10 min at 72 °C. All PCR products were detected by electrophoresis on a 2.5% (w/v) agarose gel containing GoldView Nucleic Acid Stain (an alternative to ethidium bromide, Xi’an Heart Biological Technology Co., Ltd. Xi’an, Shaanxi, China) in 1  ×  TAE buffer (pH 8.0) and were visualized under UV light.

### Sequencing of genomic DNA samples

For the samples to be sequenced, a 226-bp fragment for CYP2C19*2 and a 450-bp fragment for CYP2C19*3 were amplified using sequencing primers (Table 1). The PCR products were sequenced by the Beijing Genomic Institute (Beijing, China).

### Sensitivity of RT-LAMP assay

The sensitivities were assessed using the optimized RT-LAMP assay with 10-fold serial dilutions of plasmids. A standard curve was generated by plotting T_peek_ values against relative input copy numbers.

### Evaluation of RT-LAMP using peripheral blood samples

To evaluate the feasibility of RT-LAMP in clinical applications, 100 peripheral blood samples were tested with Tris-EDTA buffer as the negative control. All of the samples were also further verified by conventional PCR and direct sequencing.

## Additional Information

**How to cite this article**: Zhang, C. *et al*. Establishment and application of a real-time loop-mediated isothermal amplification system for the detection of *CYP2C19* polymorphisms. *Sci. Rep*. **6**, 26533; doi: 10.1038/srep26533 (2016).

## Supplementary Material

Supplementary Information

## Figures and Tables

**Table 1:** Primer sequences used for sequencing, RT-LAMP and conventional PCR reactions.

Allele	Application	Primer	Sequence
CYP2C19*2	Sequencing	*2-seq-F	5′-CAACCAGAGCTTGGCATATTG-3′
		*2-seq-R	5′-CAATAAAGTCCCGAGGGTTGT-3′
	RT-LAMP	*2-FIP-A	5′-TGGGAAATAATCAATG-CTATACCTTTATTAAATGC-3′
		*2-FIP-G	5′-CGGGAAATAATCAATG-CTATACCTTTATTAAATGCT-3′
		*2-BIP-A	5′-AGGAACCCATAACAAATTACTT-GTTCTTTTACTTTCTCC-3′
		*2-BIP-G	5′-GGGAACCCATAACAAATTACTT-GTTCTTTTACTTTCTCC-3′
		*2-F3	5′-CAGAGCTTGGCATATTGTAT-3′
		*2-B3	5′-TAAAGTCCCGAGGGTTGTTG-3′
	Conventional PCR	*2-F	5′ ACAACCAGAGCTTGGCATATTGT-3′
		*2-R-A	5′- GGTTTTTAAGTAATTTGTTATGGGTTGCT-3′
		*2-R-G	5′- TTTTTAAGTAATTTGTTATGGGTTGCC-3′
CYP2C19*3	Sequencing	*3-seq-F	5′-AGGCTGTAATTGTTAATTCGAGA-3′
		*3-seq-R	5′-TGTACTTCAGGGCTTGGTCA-3′
	RT-LAMP	*3-FIP-A	5′-TCAGGGGGTGCTTACAATCCTG-AGATCAGCAATTTCTTAACTTGA-3′
		*3-FIP-G	5′-CCAGGGGGTGCTTACAATCCTG-AGATCAGCAATTTCTTAACTTGA-3′
		*3-BIP-A	5′-AATCCAGGTAAGGCCAAGTTT-TGTACTTCAGGGCTTGGTC-3′
		*3-BIP-G	5′-GATCCAGGTAAGGCCAAGTTT-TGTACTTCAGGGCTTGGTC-3′
		*3-F3	5′-ATTTTCCAGAAACGTTTCGA-3′
		*3-B3	5′-TGTCTAGGCAAGACTGTAGT-3′
	Conventional PCR	*3-F	5′- TGTGCTCCCTGCAATGTGAT-3′
		*3-R-A	5′- AAAAAACTTGGCCTTACCTGGAAT-3′
		*3-R-G	5′- AAAAACTTGGCCTTACCTGGAAC-3′

**Table 2:** Gene test results and frequency of 200 clinical samples of *CYP2C19* alleles (type-specific concordance among RT-LAMP, conventional PCR and direct sequencing).

RT-LAMP	Conventional PCR (n = 100)						Total	Sequencing (n = 100)						Total	Agreement (%)	Frequency (%)
	*1/*1	*2/*2	*3/*3	*1/*2	*1/*3	*2/*3		*1/*1	*2/*2	*3/*3	*1/*2	*1/*3	*2/*3			
*1/*1	51	0	0	0	0	0	51	51	0	0	0	0	0	51	100%	51% (F1)
*2/*2	0	6	0	0	0	0	6	0	6	0	0	0	0	6	100%	6% (F2)
*3/*3	0	0	2	0	0	0	2	0	0	2	0	0	0	2	100%	2% (F3)
*1/*2	0	0	0	35	0	0	35	0	0	0	35	0	0	35	100%	35% (F4)
*1/*3	0	0	0	0	4	0	4	0	0	0	0	4	0	4	100%	4% (F5)
*2/*3	0	0	0	0	0	2	2	0	0	0	0	0	2	2	100%	2% (F6)
Total	51	6	2	35	4	2	100	51	6	2	35	4	2	100	100%	100%

*1/*1: CYP2C19*2 G681G type, CYP2C19*3 G636G type; *2/*2: CYP2C19*2 A681A type, CYP2C19*3 G636G type; *3/*3: CYP2C19*2 G681G type, CYP2C19*3 A636A type, *1/*2: CYP2C19*2 G681A type, CYP2C19*3 G636G type; *1/*3: CYP2C19*2 G681G type, CYP2C19*3 G636A type; *2/*3: CYP2C19*2 G681A type, CYP2C19*3 G636A type.
