# Supplementary material for: Establishment and application of a real-time loop-mediated isothermal amplification system for the detection of CYP2C19 polymorphisms
Source: Sci Rep. 2016 Jun 1;6:26533. doi: 10.1038/srep26533 (PMC4887897; doi:10.1038/srep26533)

Supplementary Materials Information

Title: Establishment and application of a real-time loop-mediated isothermal amplification system for the detection of *CYP2C19* polymorphisms

Authors: Chao Zhang[1], Yao Yao[2], Juan-Li Zhu[2], Si-Nong Zhang[1], Shan-Shan Zhang[1], Hua Wei[1], Wen-Li Hui[1, 2], and Ya-Li Cui[1, 2,*]

Author affiliations:

[1]College of Life Sciences, Northwest University, Xi'an, 710069, China

[2]National Engineering Research Center for Miniaturized Detection Systems, Xi'an, 710069, China

Correspondence and requests for materials should be addressed to Y. L. C. (E-mail: yalicui@nwu.edu.cn)

Supplementary Figures:

Supplementary Figure S1. Sensitivity of the RT-LAMP assay using *CYP2C19* A681A plasmid (A and B), *CYP2C19* G636G plasmid (C and D) and *CYP2C19* A636A plasmid (E and F). (A, C and E) Sensitivity of the RT-LAMP assay as monitored using the Genie II system. (B, D and F) The standard curve was generated from a dilution series of plasmid by plotting the T_{peek} versus the plasmid copy number.

Supplementary Figure 1

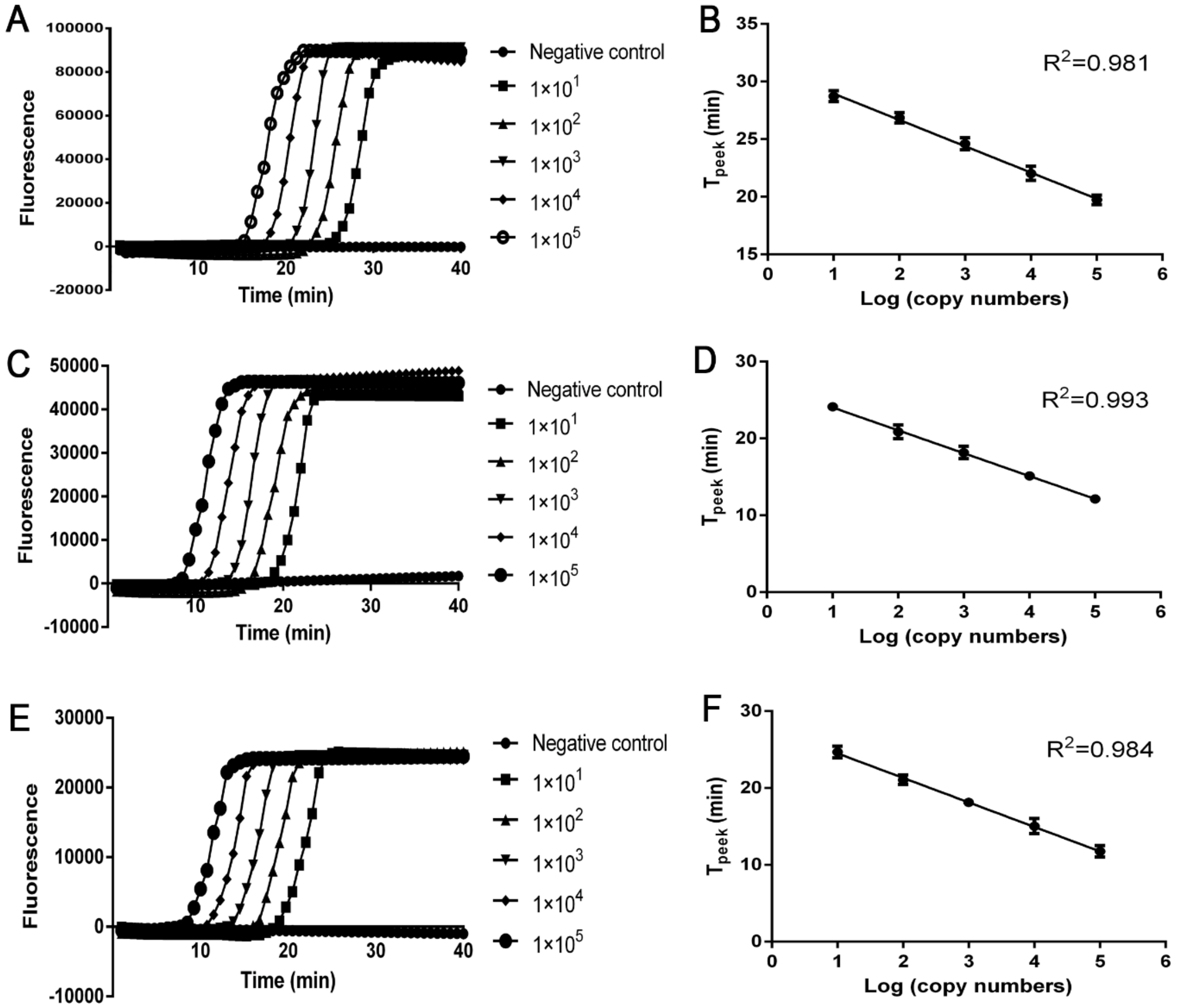

Supplement: Supplementary Information [file srep26533-s1.pdf]
